# Objective physical measures and their association with subjective functional limitations in a representative study population of older Thais

**DOI:** 10.1186/s12877-019-1093-3

**Published:** 2019-03-05

**Authors:** Orawan Prasitsiriphon, Daniela Weber

**Affiliations:** 1 College of Population Studies, Chulalongkorn University, Bangkok, 10330 Thailand; 2 Wittgenstein Centre for Demography and Global Human Capital (IIASA, VID/OEAW, WU), International Institute for Applied Systems Analysis (IIASA), 2361 Laxenburg, Austria; 3 Health Economics and Policy Division, Vienna University of Economics and Business, Welthandelsplatz 1, 1020 Vienna, Austria

**Keywords:** Handgrip strength, Usual walking speed, Functional limitations, ADL disabilities, ROC analysis

## Abstract

**Background:**

In this study, we analyzed elderly people in Thailand to identify the validity of suggested cutoff points of physical measures, handgrip strength, usual walking speed, and a composite score of both measures to predict functional limitations. Moreover, we examined whether these physical performance measures are accurate indicators of the investigated health outcomes.

**Methods:**

Using Receiver Operating Characteristics (ROC) analysis, we investigated a sample of 8272 respondents aged 60 to 79 years. All data were based on the 2009 National Health Examination Survey (NHES IV) of Thailand.

**Results:**

For males aged 60 to 69 years, handgrip strength was used as an indicator of functional limitations. The cutoff point for disabilities in the activities of daily living (ADLs) was 29.5 kg, while in other limitations it ranged from 28.7 to 31.3 kg. In contrast, usual walking speed was able to indicate ADL disabilities at 0.7 m per second (m/s). As one might expect, the cutoff points for males aged 70 to 79 years were lower than for males in the 60 to 69 age group. For females, handgrip strength was able to indicate ADL disabilities at 16.5 kg for both the 60 to 69, and 70 to 79 age groups. Likewise, walking speed was indicative of ADL disabilities at 0.6 m/s for both age groups. Interestingly, the composite measure increases the ability to detect ADL disabilities in the younger group but not in the older group. The area under the curve (AUC) of cutoffs measuring the detection power of a diagnostic test was varied, ranging from 0.535 to 0.7386.

**Conclusions:**

The cutoff points of three measures varied according to sex and type of functional limitations. Our findings also showed that physical performance measures were useful for identifying people with an increased risk of functional limitations, particularly for ADL disabilities. However, although the AUC of the cutoffs of other functional limitations were relatively low, they should be considered with caution.

**Electronic supplementary material:**

The online version of this article (10.1186/s12877-019-1093-3) contains supplementary material, which is available to authorized users.

## Background

The global trend of an increasing number of elderly people in the population is also observed in Thailand. Research shows that around 40% of elderly Thais experience at least one functional limitation in their daily activities and that limitations increase with age [1]. Care of the elderly population thus poses growing challenges for the individual, their families, and the government in Thailand.

Handgrip strength (HGS) and usual walking speed (UWS) are objective measures of overall muscle strength and physical function. Therefore, HGS and UWS are also included in most of the diagnostic criteria of sarcopenia characterized by the presence of low muscle mass and low muscle strength [2]. HGS and UWS are used to measure the particular physical abilities needed to accomplish common daily activities [3–6]. Many studies in community and clinical settings revealed an association between low HGS and a higher risk of mortality [3, 4, 7–12] and disability [5, 10]. Slow walking speed has also been associated with a greater risk of mortality [4, 6, 13–15], disability, subsequent nursing home admission [13, 14, 16], and cognitive problems [17].

Recent research in the field of geriatrics has attempted to identify people at high risk for functional limitations or frailty (i.e elevated decline in physiologic reserve and function) by determining thresholds for objective muscle-strength measurements (i.e., HGS, UWS). Although previous studies [18] indicate some consensus on cutoff values, these values are dependent on the availability of reference studies. The thresholds most frequently used to identify people with i) slow WS and sarcopenia or ii) frailty were 0.8 m/s [2] and 0.8 to 0.9 m/s [2, 19] respectively. Likewise, cutoff points to identify people with i) low HGS and sarcopenia or ii) specific disabilities were identified at 32 to 35 kg and 21 to 22 kg for males and females respectively, in Europe, America, Turkey, and some regions of Finland [20–23]. Moreover, a few studies reported cutoff values in HGS related to a specific disability [20, 24, 25]. However, these values could be different for Thais, given the differences in their physique compared with Europeans.

Several studies have shown the power of physical performance measures or composite scores to differentiate variations in functional limitations. For instance, Sieno et al. [26, 27] suggested that as a single performance measurement, UWS and HGS tests had comparable power to assess disability [27]. In another study on older women [26], UWS was found to be a better marker of poor mobility than a lower-extremity performance test comprising HGS, manipulating pegs in a pegboard, and functional reach. In terms of its power to detect disabilities in the activities of daily living (ADLs), UWS was as good a marker as the overall function measure derived from lower-extremity strength (tandem stance, chair stand test, alternate step, and get-up and go) and upper-extremity strength. This finding held despite a 3 to 5% difference between the areas under the receiver operating characteristic (ROC) curves of UWS and overall function. Guralnik et al. [28] also revealed that for predicting ADL and mobility disabilities in community-dwelling (non-institutionalized) populations, the UWS alone was nearly as good as the overall function (created by summing the test scores for standing balance, usual walking speed, and rising five times from a chair), having a 3 to 5% range of difference in areas under the curve (AUCs).

The objective of this study is to identify the validity of suggested cutoff points of physical measures (e.g., HGS, UWS, and a composite score of both measures) to project the increased likelihood of functional limitations. Using data from the 2009 National Health Examination Surveys of Thailand (NHES IV), we also investigated whether a combination of HGS and UWS can more accurately detect functional limitations in older Thais than each physical measure taken alone. As well as identifying the cutoff points for older Thai adults, this study aims to present their implications for development interventions and healthcare policies, given that cutoff points offer a simple and accurate way to identify older people at risk of disabilities*.*

## Methods

### Data and study population

The study population was drawn from the 2009 National Health Examination Survey of Thailand (NHES IV), which was the first study in Thailand to examine objective measures of mobility at a national level. A four-stage stratified probability sampling method was used to yield a nationally and regionally representative sample of the non-institutionalized population. Ultimately, a sample of 39,290 participants over the age of 15 from Bangkok and 20 provinces nationwide was obtained. The NHES IV was approved by the Ethical Review Committee for Research in Human Subjects of the Ministry of Public Health, and all participants provided written informed consent.

The sample was controlled as follows: i) the sample population was restricted to respondents aged 60 to 79 years; ii) respondents completed at least one of the physical performance tests; and iii) respondents completed the questionnaire on self-reported outcome measures, namely, Activities of Daily Living (ADLs) and Instrumental Activities of Daily Living (IADLs), upper-body limitations, lower-body limitations, and higher functional limitations. After applying these controls, 8272 respondents remained in our sample. We further excluded outliers such as those with the ability to walk 4 m in under 0.5 s (37 people). An overview of the final sample size by physical performance and self-reported outcome measurements is provided separately for males and females in Table 1.

### Measurements

#### Self-reported health outcome measurements

##### Activities of daily living (ADL)

A modified version of the Katz ADL scale with 6 items was included in the questionnaire [29]. When a participant reported at least one difficulty in basic self-care activities without assistance, including eating and drinking, dressing, bathing, using the toilet, walking across a room, and transferring from beds or chairs, the variable ADL was coded with a 1, or with a 0 if no difficulty was reported.

##### Instrumental activities of daily living (IADL)

Participants needing help with at least one instrumental activity, including using the telephone, handling money, managing medication, using transportation or driving, and housekeeping [30], were identified as having limitations in terms of performing IADLs.

##### Upper-body limitations

Respondents unable to carry heavy objects without assistance were defined as having upper-body limitations.

##### Lower-body limitations

Respondents unable either to walk at least 400 m without resting or to go up/down a flight of 10 stairs without resting were defined as having lower-body limitations.

##### Higher functional limitations

Respondents who were unable to perform at least two of the activities unassisted, such as cutting toenails, heavy housework, carrying heavy objects, and walking at least 400 m without resting were defined as having higher functional limitations.

#### Physical variables

##### Handgrip strength (HGS)

HGS was measured in kilograms (kg) using a handgrip dynamometer, Grip-D T.K.K.5401. Respondents were instructed to sit with the elbow at a 90^o^ angle with the wrist in a neutral position, keeping the upper arm tight against the trunk, and the inner lever of the dynamometer adjusted to fit the hand. Before the test, the respondents were asked if they had experienced any problems from recent surgery, injury, or other conditions of either the left or right hand within the last three months. Respondents willing to do the test and reporting no hand problems were asked to squeeze the dynamometer as hard as possible for a few seconds. Two maximum measures were recorded for each hand. The maximum HGS measure was used for this analysis.

##### Usual walking speed (UWS)

Survey participants aged at least 60 who were willing and able to do the timed walk test (walking aids were permitted), were asked to walk 4 m at their usual pace. The time to walk 4 m was recorded in seconds–in our study we measured walking speed in meters per second (m/s).

##### Overall function

A composite score of muscle strength and physical performance was constructed based on HGS and UWS. As the measurements for each test were recorded in different units, we applied sex-specific standardization with a mean of 10 and calculated a sum score.

### Demographic variables

The demographic characteristics included age, gender (male/female), body weight, body height, education, and geographic location of residence. Age corresponded to the date of birth stated on an identity card. Body weight and height were measured following a standardized protocol as a component of the physical performance measures of the NHES IV. Education was categorized into eight levels: no education, primary school, junior high school, senior high school, vocational certificate/diploma, bachelor’s degree or higher, temple education, and others. We recoded the variable education into three levels for this study: no education, primary school, and at least secondary school. The geographic area variable indicated the location of the respondent’s residence, distinguishing five regions (i.e., Bangkok, North-, Central-, Northeast-, and South Thailand). All these demographic variables were included in the analysis as potential confounders.

### Health variables

Health variables included cognitive impairment, diabetes, and hypertension, based on clinical diagnosis and self-reporting. Respondents had a blood test to determine their fasting plasma glucose (FPG), and if this was 126 mg/dl and above, the person was diagnosed with diabetes. Self-reported diabetes was determined with the question, “Has a doctor diagnosed you with this problem?” Trained physicians following a standard protocol measured respondents’ blood pressure three times using an automatic blood pressure monitor. An average of the last two brachial systolic and diastolic blood pressure readings was used for systolic and diastolic blood pressure values, if the first test gave unusually high values. Respondents were defined as having hypertension if i) they had a systolic blood pressure of 140 mmHg and above, or a diastolic pressure of 90 mmHg and above, or ii) if they were taking medication to reduce their blood pressure. Cognitive impairment was indicated following the approach used by Langa et al. [31], in which a composite score using different information from self-reported respondents was used. Specifically, we used the assessment of difficulty in two questions about memory (concentration and new learning) ranging from excellent to poor (score 1–5) and the assessment of limitations in five IADLs: managing money, taking medication, preparing hot meals, using phones, and shopping for groceries. Responses were coded according to a four-point scale where 1 = cannot, 2 = with help, 3 = can do, and 4 = never done before. We excluded the participants who reported never having done at least one instrumental activity before in 5 IADLs (*n* = 2433). Using this information, we classified respondents as cognitively impaired if they were unable to do or needed help with at least one instrumental activity in 5 IADLs and had difficulties or some difficulties with at least one memory question. Cronbach’s alpha for this cognitive impairment is 58%. These health variables were also considered as potential confounders within the analysis.

### Statistics

First, we used descriptive statistics to characterize the study participants. We then calculated sensitivity, specificity, and the AUC of the ROC curve analysis for each physical measurement (i.e., HGS and UWS) and overall function to identify the optimal cutoff values. The optimum cutoff values of each of the three physical measurements to predict the five health outcomes (i.e., ADL disabilities, IADL disabilities, upper-body limitations, lower-body limitations, and higher functional limitations) were estimated by the minimum value of (1-sensitivity)^2^ + (1-specificity)^2^ [32]. The predictive performance of the optimum cutoff values used the Delong method [33], which was implemented in the statistical software, STATA. The AUC value, statistic, and 95% confidence interval were reported to indicate how well each physical measure performed at detecting the health outcomes [34]. Moreover, logistic regressions were used to establish the strength of each of the optimal cutoff values associated with their outcomes and adjusting for potential confounders [35]. All analyses were run separately by gender (male and female) and also by age groups (60 to 69 and 70 to 79 year olds) to avoid aging effects on the five body limitations. For instance, the younger older persons in particular, could have an effect on the detected power in ADL and IADL. The sampling weights were used to make the results representative of the older Thai population. All analyses used STATA Statistical Software Release 13.

## Results

### Characteristics of samples

The sample was divided into two age groups 60 to 69 (the younger group) and 70 to 79 years (the older group) for men and women separately. The results of the characteristics were shown in the additional data (Table 1).

**Table 1:** Descriptive overview with characteristic details for each variable by age-group and gender

	Male				Female			
	60–69 years		70–79 years		60–69 years		70–79 years	
	N	Mean (95%CI)/%Share	N	Mean (95%CI)/%Share	N	Mean (95%CI)/%Share	M	Mean (95%CI)/%Share
Age	2498	64.2 (58.6–69.9)	1566	73.9 (68.6–79.3)	2559	64.1 (58.4–69.7)	1652	73.9 (68.4–79.3)
Weight	2487	60.3 (37.7–82.9)	1557	56.3 (34.4–78.2)	2552	56.1 (33.5–78.8)	1645	51.1 (29.5–72.8)
Height	2487	162.2 (150.3–174.1)	1552	160.3 (147.8–172.8)	2544	151.4 (141.1–161.7)	1619	149.5 (138.5–160.4)
% Education level	2494		1559		2556		1650	
No education		4.4		7.9		12.2		20
Primary school		78.1		80.6		79.7		76.5
At least secondary school		17.5		11.5		8.1		3.5
% Region	2498		1566		2559		1652	
Bangkok		11.2		16.5		11.2		10.1
North		19.7		19.3		18.6		20
Central		24.5		23.9		26		24.8
Northeast		32.7		27.1		32.9		31.3
South		11.9		13.1		11.3		13.8
% Cognitive impairment	2490		1430		2382		1399	
- No		94.5		90.9		88.8		82.8
- Yes		5.5		9.1		11.2		17.2
% Diabetes	2408		1508		2479		1597	
- No		86.4		85.7		80.8		82.9
- Yes		13.6		14.3		19.2		17.1
% Hypertension	2489		1565		2554		1652	
- No		56.9		48.7		55		47.5
- Yes		43.1		51.3		45		52.5
Physical performances								
HGS (kilograms)	2489	31.5 (18.4–44.5)	1561	26.5 (13.7–39.3)	2550	21.0 (12.3–29.8)	1645	18.3 (10.2–26.4)
UWS (m/s)	2453	0.8 (0.4–1.2)	1530	0.7 (0.3–1.1)	2509	0.7 (0.4–1.0)	1607	0.6 (0.3–1.0)
Overall function	2453	4.5 (2.7–6.3)	1528	3.8 (1.8–5.7)	2504	4.0 (2.3–5.7)	1604	3.4 (1.7–5.0)
Outcome								
% ADL disabilities	2471		1547		2533		1641	
- No		98.8		96.3		98.6		97
- Yes		1.2		3.7		1.4		3
% IADL disabilities	2001		1163		2088		1275	
- No		67.1		47.2		44		18.7
- Yes		32.9		52.8		56		81.3
% Upper-body limitations	2348		1430		2385		1428	
- No		88.9		76.3		75.4		53.2
- Yes		11.1		23.7		24.6		46.8
% Lower-body limitations	2291		1423		2264		1405	
- No		86.8		73.8		66		46
- Yes		13.2		26.2		34		54
% Higher functioning limitations	2101		1244		2091		1186	
- No		92.1		79.7		77.4		57.4
- Yes		7.9		20.3		22.6		42.6

*95% CI* 95% confidence interval, *HGS* handgrip strength, *UWS* usual walking speed, *ADL* activities of daily living, *IADL* instrumental activities of daily living

In men, the average age was 64.2 years for the younger, and 73.9 years for the older age group. About 5.5% of the males in the 60 to 69 age group reported cognitive impairment, 13.6% reported diabetes, and 43.1% reported hypertension, whereas 9.1% of the older males reported cognitive impairment, 14.3% diabetes, and 51.3% hypertension. Further, the 60 to 69 age group had an average HGS of 31.5 kg, UWS of 0.8 m/s, and a score of 4.5 in average overall function. Their older counterparts had an average HGS of 26.5 kg, UWS of 0.7 m/s and overall function of 3.8. Functional limitations affected ADL disabilities, IADL disabilities, upper-body limitations, lower-body limitations, and higher functional limitations by 1.2, 32.9, 11.1, 13.2, and 7.9% respectively in the younger age group, and by 3.7, 52.8, 23.7, 26.2, and 20.3% respectively in the older age group.

In women, the mean age of the younger population was 64.1 years, and 73.9 years for the older female population. The younger female group had lower strength in HGS and UWS than males (21.0 kg, and 0.7 m/s, respectively), while the older female group had a HGS of 18.3 kg and UWS of 0.6 m/s. Both groups of women reported a greater prevalence of cognitive impairment, diabetes, and hypertension than men. The younger females reported 11.2% cognitive impairment, 19.2% diabetes, 45% hypertension, 1.4% ADL disabilities, 56% IADL disabilities, 24.6% upper-body limitations, 34% lower-body limitations, and 22.6% higher functional limitations. As expected, the older group had a higher prevalence of diseases or limitations (17.2% cognitive impairment, 17.1% diabetes, 52.5% hypertension, 3% ADL disabilities, 81.3% IADL disabilities, 46.8% upper-body limitations, 54% lower-body limitations, and 42.6% higher functional limitations) than the younger group.

Physical strength decreases with increasing age and overall, women have lower physical performance than men. By age groups and sex, age groups reporting any conditions of ADL disabilities, IADL disabilities, upper-body limitations, lower-body limitations, and higher functional limitations tend to have lower mean scores in all three physical performances. Respondents with ADL disabilities were likely to be the weakest compared to those with other functional restrictions.

### Cutoff points for HGS, UWS, and overall function

#### Males

The ability of physical performance tests to accurately differentiate between elderly people with and without functional limitations in each domain is reflected by the AUC of the ROC curve analysis for men presented in Table 2. Based on the results, HGS for predicting ADL disabilities shows moderate accuracy in males aged 60 to 69 (AUC = 0.74) and low accuracy in males aged 70 to 79 (AUC = 0.69), as well as low accuracy for IADL disabilities, upper-body limitations, lower-body limitations, and higher functional limitations in both age groups (AUC = 0.61 to 0.66). Moreover, the optimal cutoff values for men’s ADL disabilities were not much different from the other outcomes except for IADL disabilities with the highest optimal cutoff values. ADL disabilities were 29.5 kg for 60 to 69 year olds and 25.3 kg for 70 to 79 year olds, while IADL disabilities were 31.3 kg for 60 to 69 year olds and 26.4 kg for 70 to 79 year olds.

**Table 2:** Cutoff value of HGS, UWS, and overall function with self-reported ADL disabilities, IADL disabilities, upper-body limitations, lower-body limitations, and higher functional limitations by gender

	Male				Female			
	CP	Sn	Sp	AUC (95%CI)	CP	Sn	Sp	AUC (95%CI)
Handgrip strength								
60–69 years								
ADL disabilities	29.5	0.87	0.61	0.74 (0.740–0.744)	16.5	0.59	0.85	0.72 (0.714–0.720)
IADL disabilities	31.3	0.63	0.60	0.62 (0.616–0.618)	21.5	0.58	0.55	0.57 (0.566–0.567)
Upper-body limitations	28.7	0.58	0.70	0.64 (0.634–0.637)	20.8	0.56	0.55	0.55 (0.553–0.554)
Lower-body limitations	30.0	0.61	0.62	0.62 (0.616–0.618)	20.8	0.58	0.58	0.58 (0.576–0.578)
Higher functional limitations	30.0	0.59	0.62	0.61 (0.605–0.608)	20.8	0.57	0.55	0.56 (0.56–0.564)
70–79 years								
ADL disabilities	25.3	0.80	0.57	0.69 (0.685–0.689)	16.5	0.65	0.69	0.67 (0.665–0.670)
IADL disabilities	26.4	0.64	0.64	0.64 (0.636–0.638)	19.2	0.61	0.57	0.59 (0.591–0.593)
Upper-body limitations	25.4	0.64	0.62	0.63 (0.632–0.634)	19.1	0.65	0.50	0.58 (0.577–0.579)
Lower-body limitations	25.5	0.63	0.63	0.63 (0.626–0.628)	18.5	0.56	0.59	0.57 (0.573–0.575)
Higher functional limitations	25.4	0.67	0.64	0.66 (0.655–0.658)	19.1	0.65	0.52	0.59 (0.584–0.586)
Usual walking speed								
60–69 years								
ADL disabilities	0.7	0.84	0.59	0.72 (0.713–0.720)	0.6	0.56	0.82	0.69 (0.687–0.694)
IADL disabilities	0.9	0.84	0.23	0.53 (0.531–0.532)	0.8	0.68	0.47	0.58 (0.574–0.575)
Upper-body limitations	0.8	0.54	0.60	0.57 (0.570–0.573)	0.7	0.76	0.43	0.59 (0.590–0.591)
Lower-body limitations	0.8	0.57	0.61	0.59 (0.592–0.594)	0.7	0.76	0.45	0.60 (0.603–0.604)
Higher functional limitations	0.8	0.56	0.60	0.58 (0.578–0.581)	0.7	0.78	0.43	0.60 (0.600–0.602)
70–79 years								
ADL disabilities	0.6	0.80	0.79	0.80 (0.796–0.801)	0.6	0.76	0.67	0.71 (0.712–0.717)
IADL disabilities	0.8	0.68	0.47	0.58 (0.576–0.578)	0.7	0.83	0.29	0.56 (0.558–0.560)
Upper-body limitations	0.7	0.78	0.46	0.62 (0.617–0.619)	0.7	0.84	0.26	0.55 (0.549–0.550)
Lower-body limitations	0.7	0.72	0.45	0.58 (0.581–0.583)	0.7	0.83	0.25	0.54 (0.540–0.541)
Higher functional limitations	0.7	0.79	0.46	0.63 (0.627–0.629)	0.7	0.84	0.25	0.54 (0.542–0.544)
Overall function								
60–69 years								
ADL disabilities	3.5	0.83	0.88	0.86 (0.854–0.860)	3.2	0.66	0.83	0.74 (0.739–0.746)
IADL disabilities	4.3	0.52	0.70	0.61 (0.609–0.611)	4.2	0.66	0.50	0.58 (0.582–0.583)
Upper-body limitations	4.3	0.62	0.64	0.63 (0.630–0.632)	4.0	0.60	0.56	0.58 (0.579–0.580)
Lower-body limitations	4.2	0.61	0.64	0.63 (0.628–0.630)	4.0	0.60	0.59	0.59 (0.594–0.595)
Higher functional limitations	4.3	0.61	0.64	0.63 (0.624–0.627)	3.7	0.48	0.70	0.59 (0.593–0.595)
70–79 years								
ADL disabilities	2.9	0.77	0.84	0.80 (0.800–0.805)	3.0	0.68	0.71	0.70 (0.693–0.698)
IADL disabilities	3.7	0.59	0.71	0.65 (0.653–0.655)	3.5	0.58	0.60	0.59 (0.585–0.588)
Upper-body limitations	3.5	0.62	0.72	0.67 (0.664–0.666)	3.5	0.61	0.55	0.58 (0.577–0.579)
Lower-body limitations	3.7	0.66	0.64	0.65 (0.648–0.651)	3.4	0.56	0.61	0.59 (0.585–0.587)
Higher functional limitations	3.5	0.63	0.72	0.67 (0.673–0.675)	3.6	0.71	0.45	0.58 (0.579–0.581)

*CP* cutoff point, *S*_*n*_ sensitivity, *S*_*p*_ specificity, *AUC* area under curve, *ADL* activities of daily living, *IADL* instrumental activities of daily living

**Table 3:** Odd ratios for ADL disabilities, IADL disabilities, upper-body limitations, lower-body limitations, and higher functional limitations according to HGS, UWS, and overall function by gender

Variable	Male			Female		
	Model 1	Model 2	Model 3	Model 1	Model 2	Model 3
	OR	OR	OR	OR	OR	OR
Handgrip strength						
60–69 years						
ADL disabilities	17.25***	20.01***	20.48***	5.90***	6.70***	3.39*
IADL disabilities	1.84***	1.77***	1.45**	1.43***	1.73***	1.70***
Upper-body limitations	2.85***	2.79***	2.69***	1.40*	1.61***	1.62***
Lower-body limitations	2.33***	2.25***	2.19***	1.90***	2.15***	2.24***
Higher functional limitations	2.34***	2.27***	2.34***	1.62***	1.85***	1.91***
70–79 years						
ADL disabilities	10.74**	12.01**	22.80***	3.05***	3.10***	2.59*
IADL disabilities	2.26***	2.18***	2.35**	2.01***	2.05***	1.75**
Upper-body limitations	3.41***	3.61***	3.60***	1.70***	1.86***	1.34*
Lower-body limitations	2.12***	2.22***	1.93***	1.78***	1.89***	1.32*
Higher functional limitations	2.13***	2.12***	2.65***	1.75***	1.84***	1.31
Usual walking speed						
60–69 years						
ADL disabilities	6.92**	6.32**	5.77*	5.66***	5.33***	21.77***
IADL disabilities	1.33	1.17	1.08	1.77***	1.38***	1.35**
Upper-body limitations	1.72**	1.80***	2.03***	2.17***	1.92***	2.07***
Lower-body limitations	2.03***	2.15***	2.29***	2.45***	2.18***	2.21***
Higher functional limitations	1.81*	1.88***	2.35***	2.46***	2.06***	2.31***
70–79 years						
ADL disabilities	20.09***	29.67***	25.96***	4.80***	4.94***	2.45*
IADL disabilities	1.12	1.14	1.05	1.95***	1.69***	1.32
Upper-body limitations	2.81***	2.88***	2.78***	1.69***	1.57***	1.25
Lower-body limitations	1.89***	1.94***	1.97***	1.55***	1.48**	1.21
Higher functional limitations	2.93***	2.96***	2.69***	1.63**	1.54**	1.29
Overall function						
60–69 years						
ADL disabilities	46.60***	51.62***	49.81***	8.01***	8.16***	8.56***
IADL disabilities	1.91***	1.89***	1.80***	1.72***	1.76***	1.70***
Upper-body limitations	2.64***	2.66***	2.77***	1.76***	1.87***	1.93***
Lower-body limitations	2.59***	2.56***	2.71***	2.15***	2.22***	2.25***
Higher functional limitations	2.73***	2.70***	3.29***	2.18***	2.29***	2.64***
70–79 years						
ADL disabilities	14.29***	14.48***	18.88***	4.04***	4.02***	3.15*
IADL disabilities	1.58**	1.71***	1.60**	1.83**	1.70**	1.44
Upper-body limitations	2.23***	2.20***	2.19***	2.02***	2.06***	1.58**
Lower-body limitations	1.70**	1.73***	1.58**	2.17***	2.23***	1.63**
Higher functional limitations	1.72**	1.65**	1.68**	1.85***	1.83***	1.42*

*Notes*: Model 1 adjusted for age, height, and weight; Model 2 adjusted for age, height, weight, education groups, and geographic region; Model 3 adjusted for age, height, weight, education groups, and geographic region, cognitive problems, diabetes, and hypertension; *OR* Odd ratio, *ADL* activities of daily living, *IADL* instrumental activities of daily living

* *p* < 0.05, ** *p* < 0.01, *** *p* < 0.001

Using UWS as an indicator enabled us to differentiate between males with and without ADL disabilities with moderate accuracy for younger participants (AUC = 0.72) and good accuracy for older males (AUC = 0.80). Interestingly, it was not possible to discriminate between elderly people with and without functional limitations in the other outcomes except in terms of IADL disabilities and lower-body limitations in the older group. Furthermore, the optimal cutoff values for men’s ADL disabilities were lower than for the other outcomes. ADL disabilities were 0.7 m/s for the 60 to 69 age group and 0.6 m/s for the 70 to 79 age group, while the other outcomes ranged between 0.8 and 0.9 m/s for the younger groups, and between 0.7 and 0.8 m/s for the older groups.

The ability to accurately differentiate in terms of overall function was good for ADL disability (AUC = 0.86) and low for the other outcomes (AUC = 0.61 to 0.63) in males aged 60 to 69 years. In the older group, it also shows good accuracy in predicting ADL disabilities (AUC = 0.80), while accuracy for the other outcomes is low (AUC = 0.65 to 0.67). The optimal scores vary for all outcomes: in ADL disabilities, 3.5 for 60 to 69 year olds, 2.9 for 70 to 79 year olds, and in other outcomes, respectively 4.2 to 4.3 and 3.5 to 3.7.

#### Females

The ability of physical performance tests to accurately project women’s health outcomes is presented in Table 3. HGS shows moderate (AUC = 0.72) and low accuracy (AUC = 0.67) for predicting ADL disabilities in both the younger and older group. HGS fails to detect the other health outcomes (AUC < 0.6) in both age groups. The optimal cutoff values for ADL disabilities are 16.5 kg for both the 60 to 69 age group and the 70 to 79 age group. The cutoff ranges for the other outcomes are 20.8 to 21.5 kg in younger females and 18.5 to 19.2 kg in older females.

By using UWS as a measure, we were able to detect females aged 60 to 69 years with and without ADL disabilities (AUC = 0.69), as well as lower-body limitations (AUC = 0.60) and higher functional limitations (AUC = 60). UWS also differentiates for ADL disabilities in the older group with moderate accuracy (AUC = 0.71). The cutoff values of UWS in identifying IADL disabilities and upper-body limitations failed (AUC = 0.55–0.59). The cutoff points of UWS in 60 to 69 year olds were 0.6 m/s for ADL disabilities, 0.8 m/s for IADL disabilities, and 0.7 m/s for others. For the older group, the cutoff points were 0.6 m/s for ADL disabilities and 0.9 m/s for the other outcomes.

Overall function shows similar results to those of HGS and has a moderate accuracy in predicting ADL disabilities among both younger (AUC = 0.74) and older females (AUC = 0.70), but it failed to accurately predict the other outcomes. The optimal cutoff rate in overall function is 3.2 for 60 to 69 year olds and 3.0 for 70 to 79 year olds. The other outcomes range between 3.7 and 4.2 for the younger group, and between 3.4 and 3.5 for the older one.

The ROC curves of the handgrip strength, usual walking speed and overall function to detect ADL disability, IADL disability, upper-body functions, lower-body functions and higher functional limitations among Thai elderly people by age groups and sexes are shown in Additional file 1: Figures S1-S5.

### Associations between HGS, UWS, and overall function, and the prevalence of five functional limitations

The results of the investigation of the relationship between good physical performance, which was identified by the previously defined cutoff values, and health outcomes for men and women are presented in Table 3. There are many significant associations between the three physical performance measurements (HGS, UWS, and overall function) and the health outcomes for both age groups. For both sexes and both age groups, the adjusted-odds ratios of physical tests to ADL disabilities were higher than in other functional restrictions. These results remained stable following the addition of potential confounders such as education, region of residence, cognitive impairment, diabetes, and hypertension. However, we found that UWS is not significant for IADL disabilities in the two male groups (models 1 to 3). In the younger and older female population, there was a relationship with being functionally limited in each outcome of the physical performance tests. This was however not the case in the older female group when controlling for all confounders (model 3). UWS is therefore not a significant indicator for all functional restrictions except ADL disabilities, while HGS and overall function are not significant indicators for IADL disabilities and higher functional limitations, respectively.

## Discussion

This analysis aimed to identify the validity of the suggested cutoff points of physical measures (HGS, UWS, and overall function) to predict functional limitations (i.e., ADL disabilities, IADL disabilities, upper-body limitations, lower-body limitations, and higher functional limitations). Our results show that the predictive power of the cutoff points of the three physical measures varied according to health outcomes and sex. For instance, the HGS cutoff points showed a significantly higher predictive power for men than for women. We also found that not all performance measurements were good indicators of functional limitations. We further found that overall function had more power to detect ADL disabilities than physical tests alone.

Some variations in the norms [36] and risk thresholds of performing tasks at different difficulty levels [21–23, 36] among older adults are also supported by our results. The mean value of UWS for elderly American and Italian community-dwellers ranges from 1.03 to 1.07 m/s, whereas usual walking speeds of 0.60 to 1.00 m/s could be predictive of future risk factors for adverse health outcomes in various populations [28, 37–39]. For HGS, the mean maximum range was from 31.3 to 41.0 kg and 19.2 to 25.0 kg respectively, for older male and female adults in Western countries. HGS at 35 kg in men and 22 kg in women showed a risk of weakness [21] and HGS at 32 to 33 kg in men and 19 to 21 kg in women showed a risk of impaired mobility [22, 23, 36]. A comparison with our findings reveals that, when considering available cutoff values (AUC ≥ 60), the cutoff points for UWS provided a value of less 0.8. The HGS cutoff points showed a slightly lower value in the younger age group and an even lower value for ADL disabilities.

ADL disabilities in our study occur at the lowest values of each physical performance, which is consistent with the results of a previous study [40]. Young et al. (2010) showed that the mean value of UWS was the highest for older women unable to do heavy housework, followed by those unable to bathe, do shopping, walk up 10 steps, dress, and get out of bed. This is consistent with a recent study showing that the cutoff points were associated with outcomes [41] and is also confirmed by our own findings. Our findings suggest that the inability to perform tasks of daily living is more likely to increase with increasing weakness in muscular strength than IADL tasks or other functional limitations.

According to single physical measures in our study, the HGS test provided cutoff values (AUC ≥ 60) for all functional limitations in both the younger and older age groups of elderly Thai men and for ADL disabilities in elderly Thai women. UWS provided cutoff values for ADL disabilities in elderly males and females of both age groups, upper-body and higher-body functional limitations in males aged 70 to 79 years, and lower-body and higher-body functional limitations in females aged 60 to 69. Moreover, these cutoff values were significantly associated with functional limitations, although potential confounding factors were controlled for. We also found that the combination of HGS and UWS tests as representative of overall function appeared to better differentiate between with- and without- ADL disabilities in both age groups and sexes than single physical performance measures. Our findings indicate that combined performance measures could increase the ability to predict ADL disabilities among elderly Thais. However, UWS was better to identify disabilities in the older Thai group.

The present study first addressed the cutoff values to assess functional limitations of performance-based measurements for two age groups of elderly Thai people from a nationwide representative sample. These cutoff points have important implications in terms of identifying the risk of disabilities in elderly Thais, which would place a heavy burden on both families and society with respect to healthcare cost and assistance needs. Early detection of disabilities provides the greatest advantages for direct preventive interventions. Nevertheless, as the present study is limited by its cross-sectional nature and health is a dynamic concept, we are unable to investigate the association between physical-based measurement and functional limitations over time. In addition, as the age range of the sample population is limited to between 60 and 79 years, Thais aged 80 years and above are not included. Further studies should assess the association between the changes in individual physical performance and functional limitations that include the oldest old Thais.

Moreover, AUC which was used in our study to measure the detection power of a diagnostic test, varied in performance according to the type of functional limitations in question, ranging from 0.53 to 0.86, and was thus found not to be suitable for clinical use except to determine ADL disabilities [42]. Although previous studies suggested that the self-reporting approach including ADLs, IADLs, mobility performance, and objective physical performance tests could potentially predict health status [2, 19, 20], the interview and examination procedures of the NHES IV of Thailand took place at local health centers, schools, or temples in the community, which may have been a problem for more severely disabled people. Therefore, further attempts to investigate physical performance scores in a clinical setting with a heterogeneous level of disability of Thai elderly people are needed to confirm our findings.

## Conclusions

To sum up, the cutoff points of three measures of HGS, UWS, and overall function in our results varied according to the type of functional limitations and the sex of the subject. These values suggested that there is potential to distinguish men and women presenting a higher risk of ADL and other functional limitations (IADL disabilities, upper-body limitations, lower-body limitations, and higher functional limitations). However, as the AUC of the cutoffs of other functional limitations were relatively low, they should be considered with caution. Interestingly, overall function is more useful than HGS and UWS alone for detecting ADL disabilities in Thai elderly people. Future studies should examine the cutoff points in a clinical setting with a heterogeneous level of disabilities of elderly Thais and include the results in the next NHES survey.

## Additional file

Additional file 1: **Figure S1-S5.** (DOCX 27715 kb)

## Abbreviations

ADLs: Activities of daily living
AUCs: Areas under the curve
FPG: Fasting plasma glucose
HGS: Handgrip strength
IADLs: Instrumental activities of daily living
NHES IV: National health examination surveys of Thailand
ROC: Receiver operating characteristic
UWS: Usual walking speed
