# Supplementary material for: Objective physical measures and their association with subjective functional limitations in a representative study population of older Thais
Source: BMC Geriatr. 2019 Mar 5;19:73. doi: 10.1186/s12877-019-1093-3 (PMC6402119; doi:10.1186/s12877-019-1093-3)
Supplement: Supplementary file 1 — Figure S1-S5. (DOCX 27715 kb) [file 12877_2019_1093_MOESM1_ESM.docx]

**Figure S1** Receiver operating characteristic (ROC) curve of the handgrip strength, usual walking speed and overall function to detect ADL disability among Thai elderly people by age groups and sexes.

**Handgrip strength (HGS)**

**60-69 years old**


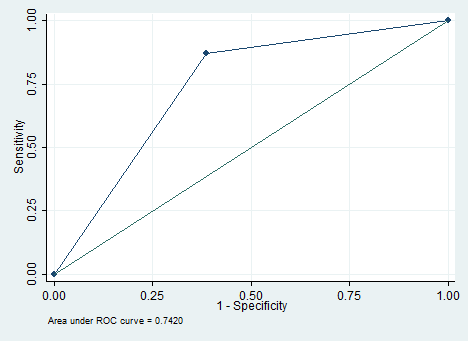

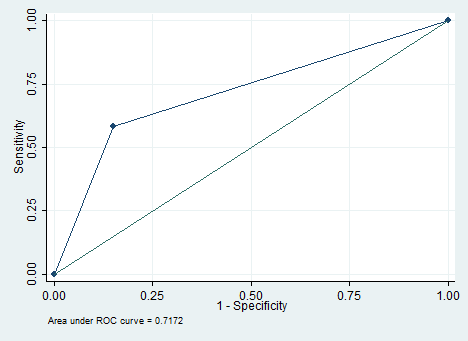


**70-79 years old**


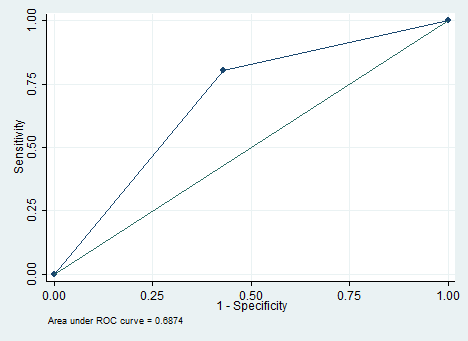

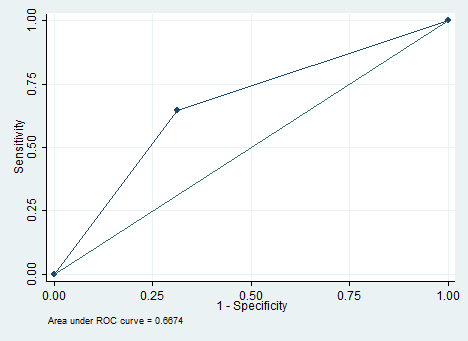


**Usual walking speed (UWS)**

**60-69 years old**


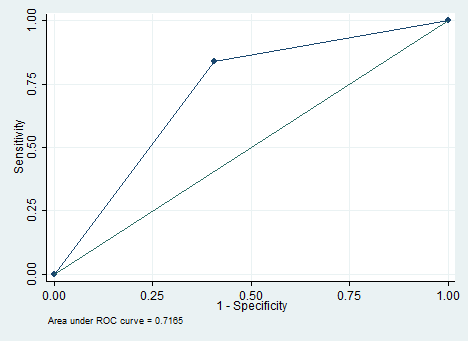

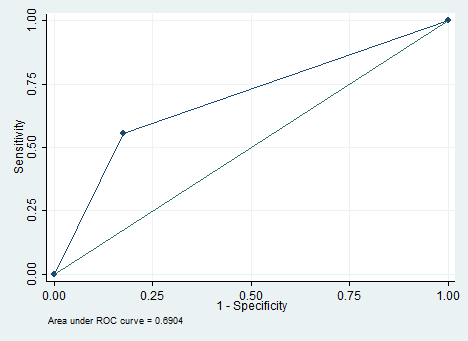


**70-79 years old**


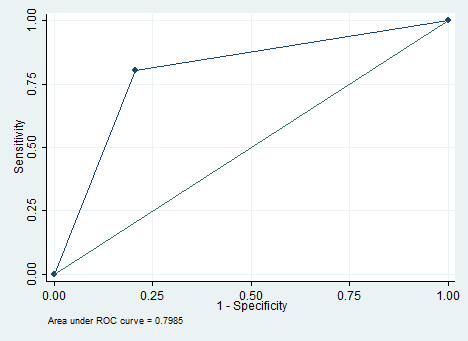

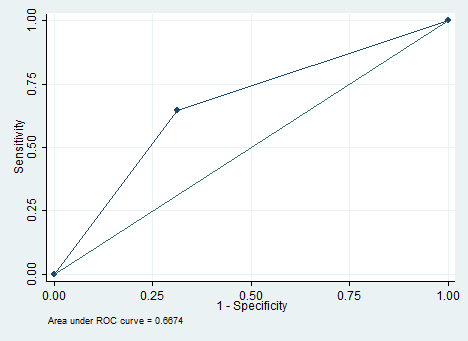


**Overall functions**

**60-69 years old**


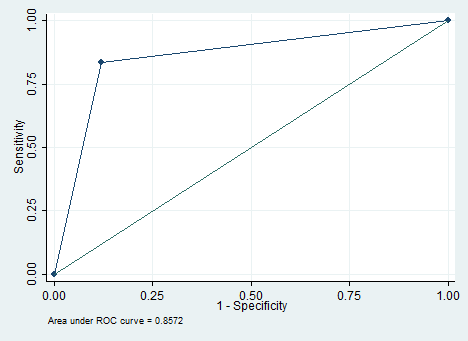

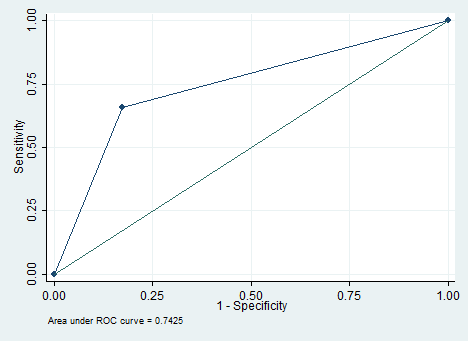


**70-79 years old**


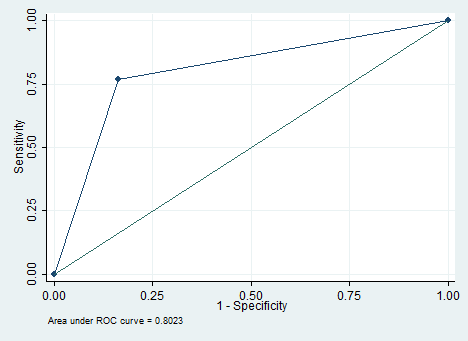

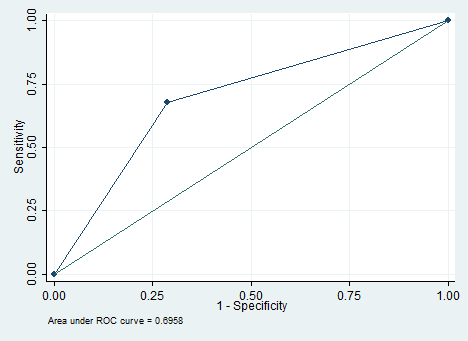


**Figure S2** Receiver operating characteristic (ROC) curve of the handgrip strength, usual walking speed and overall function to detect IADL disability among Thai elderly people by age groups and sexes.

**Handgrip strength (HGS)**

**60-69 years old**


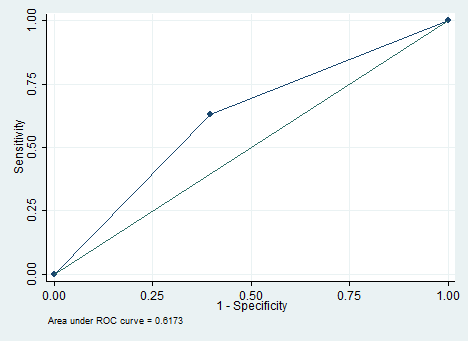

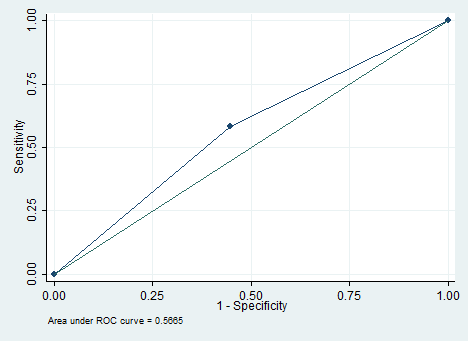


**70-79 years old**


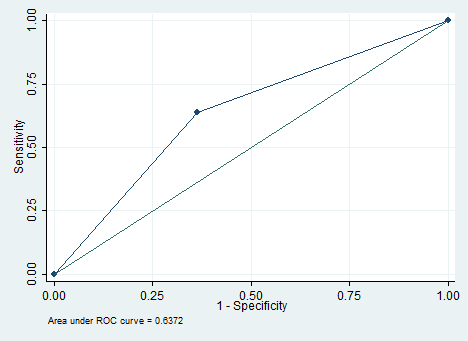

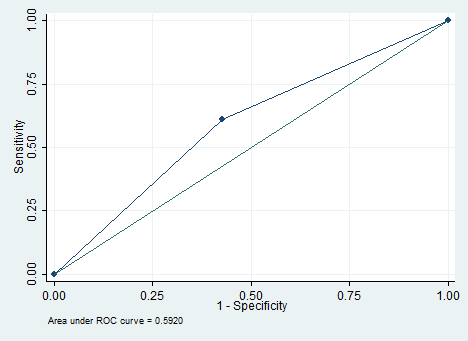


**Usual walking speed (UWS)**

**60-69 years old**


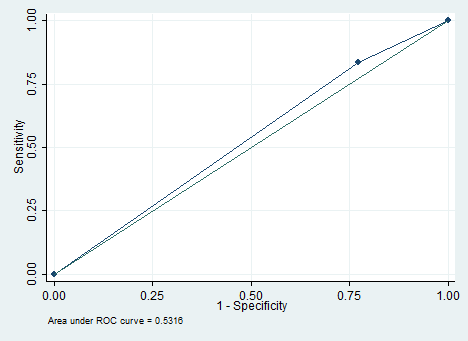

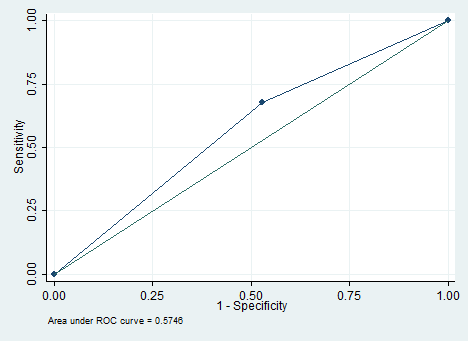


**70-79 years old**


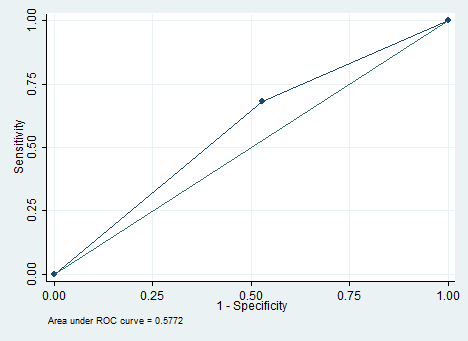

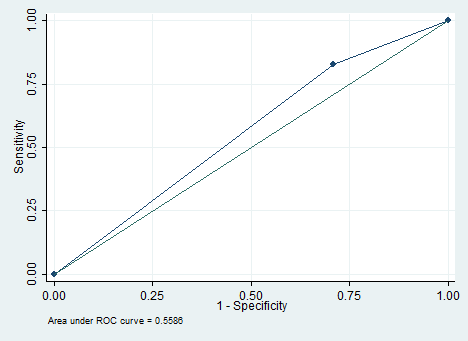


**Overall functions**

**60-69 years old**


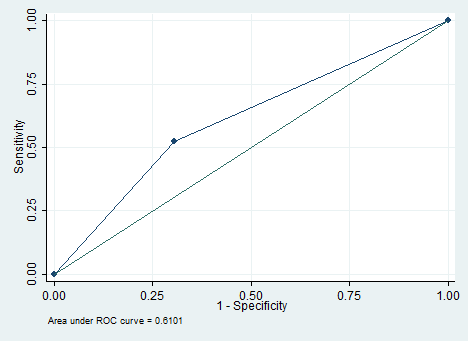

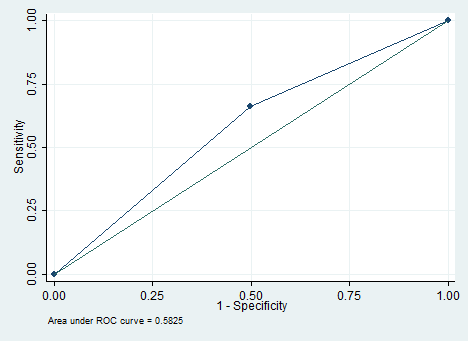


**70-79 years old**


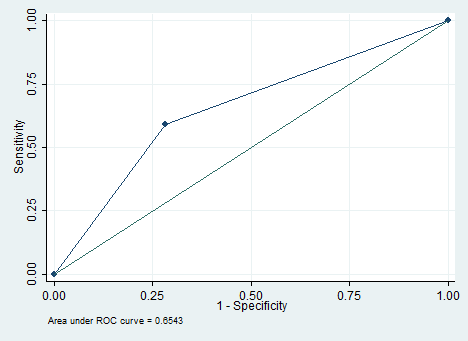

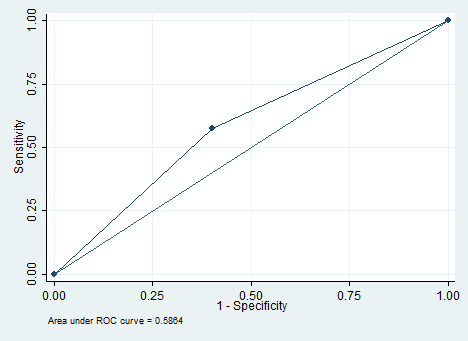


**Figure S3** Receiver operating characteristic (ROC) curve of the handgrip strength, usual walking speed and overall function to detect upper-body functions among Thai elderly people by age groups and sexes.

**Handgrip strength (HGS)**

**60-69 years old**


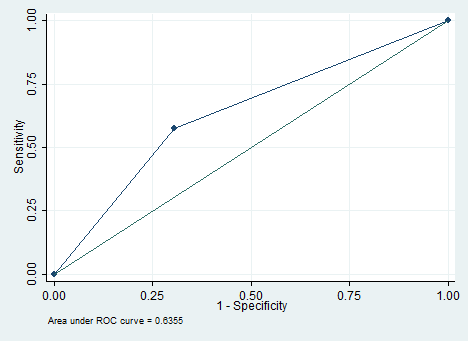

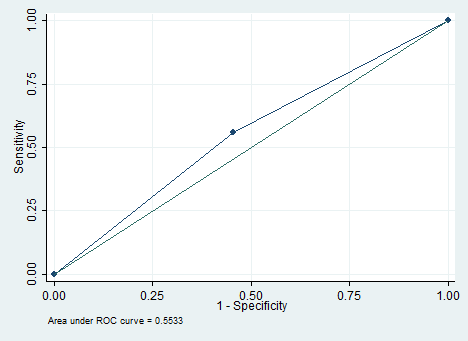


**70-79 years old**


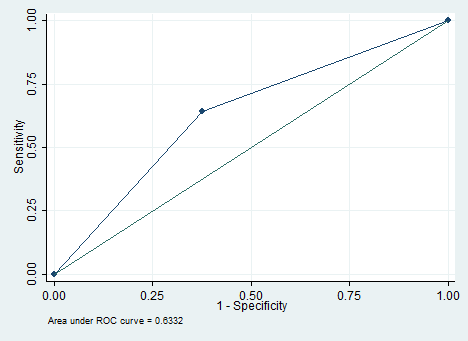

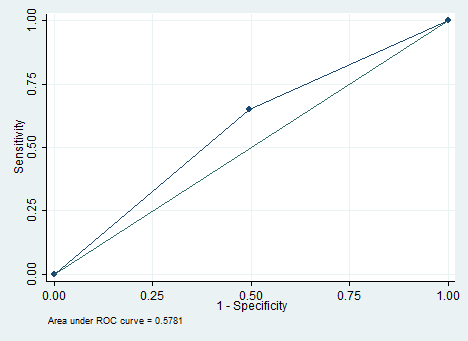


**Usual walking speed (UWS)**

**60-69 years old**


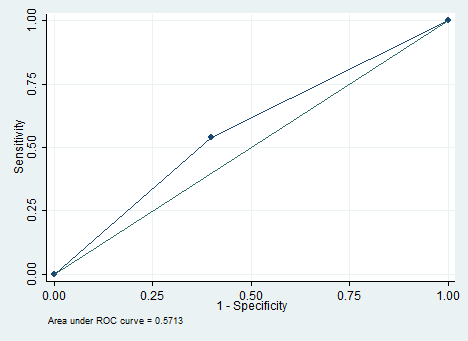

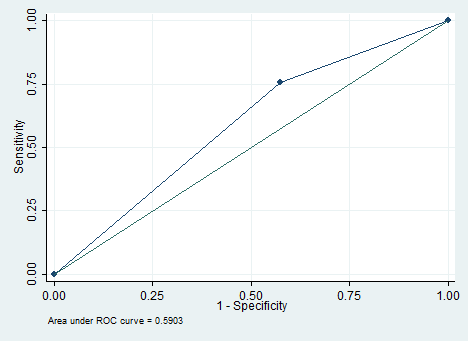


**70-79 years old**


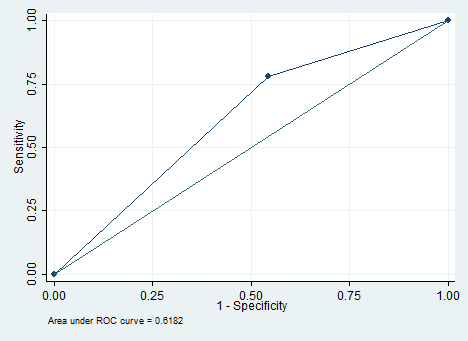

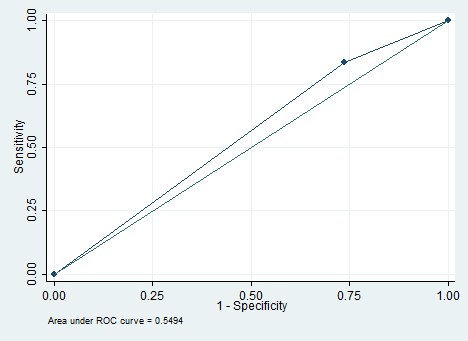


**Overall functions**

**60-69 years old**


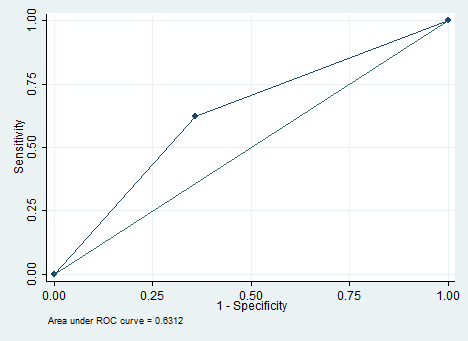

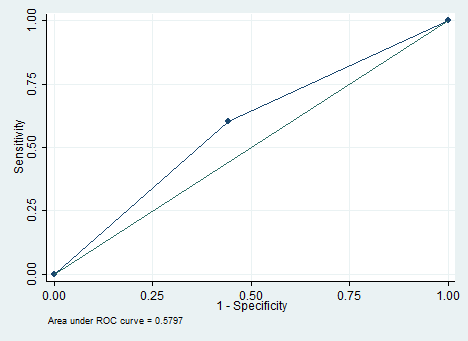


**70-79 years old**

**
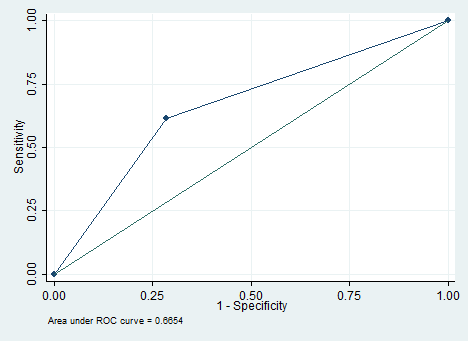

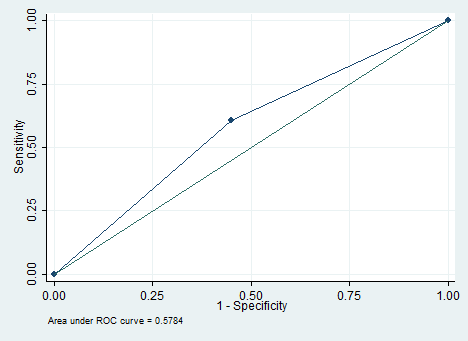
**

**Figure S4** Receiver operating characteristic (ROC) curve of the handgrip strength, usual walking speed and overall function to detect lower-body functions among Thai elderly people by age groups and sexes.

**Handgrip strength (HGS)**

**60-69 years old**


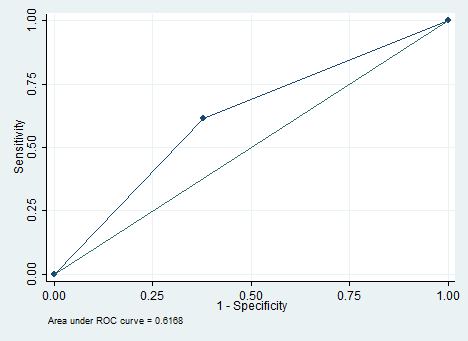

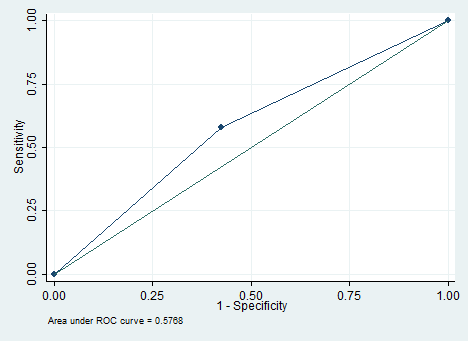


**70-79 years old**


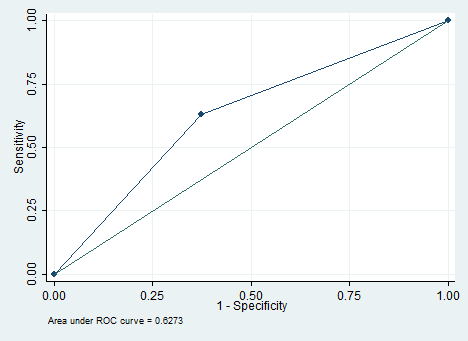

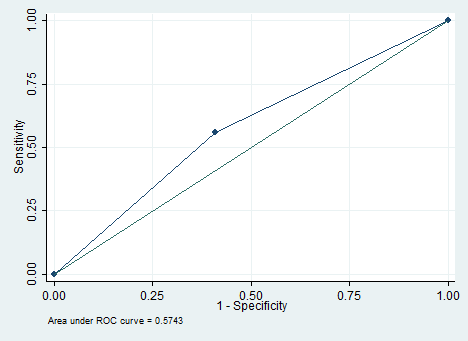


**Usual walking speed (UWS)**

**60-69 years old**


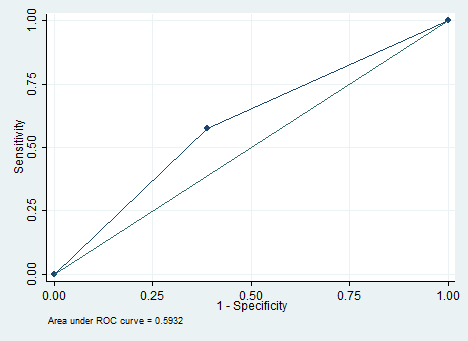

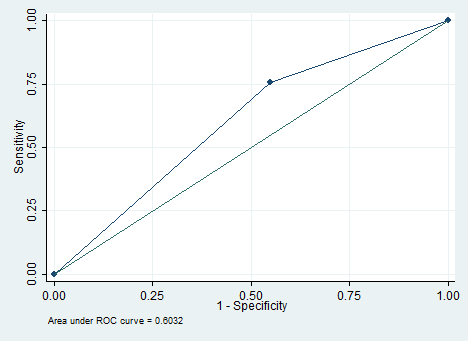


**70-79 years old**


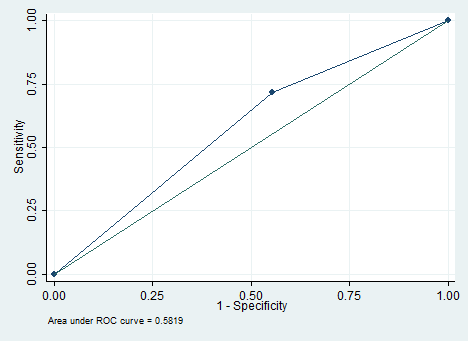

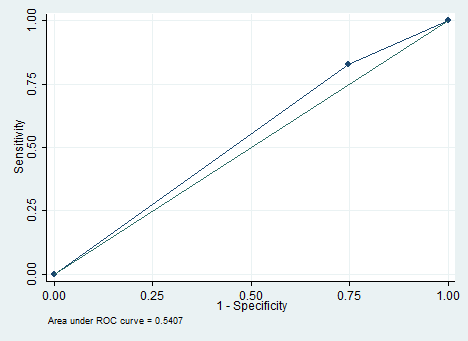


**Overall functions**

**60-69 years old**


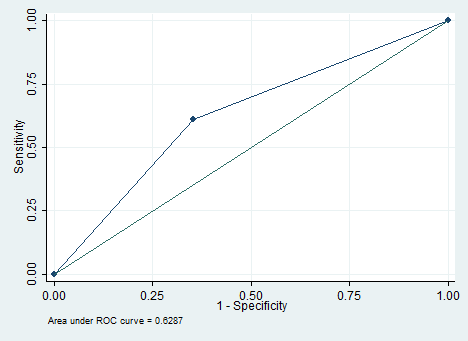

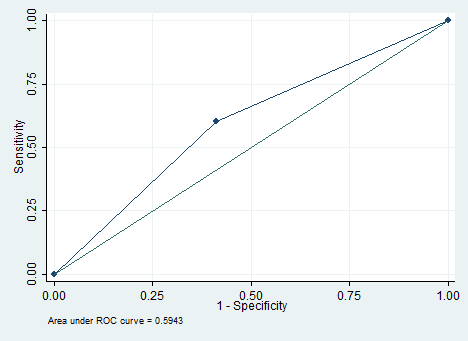


**70-79 years old**

**
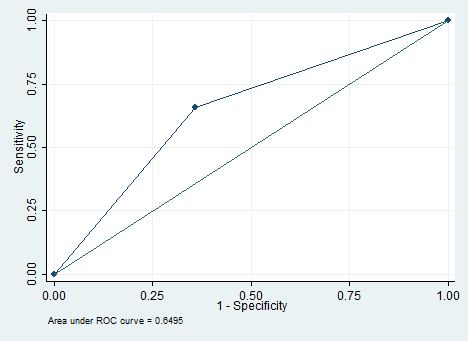

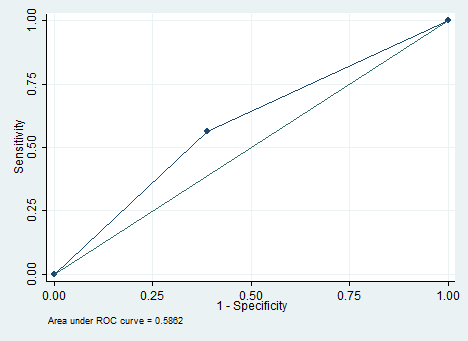
**

**Figure S5** Receiver operating characteristic (ROC) curve of the handgrip strength, usual walking speed and to detect higher functional limitations among Thai elderly people by age groups and sexes.

**Handgrip strength (HGS)**

**60-69 years old**


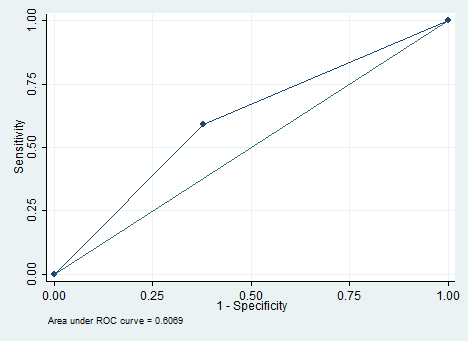

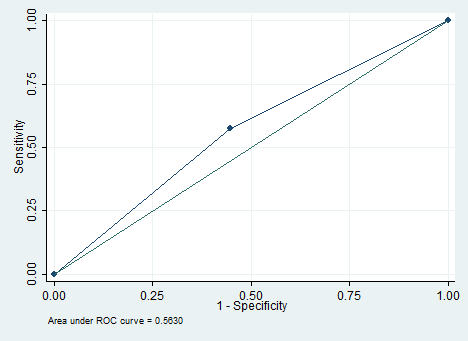


**70-79 years old**


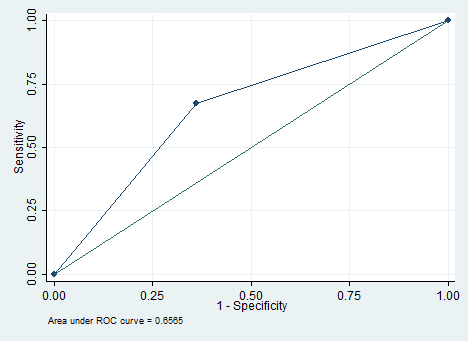

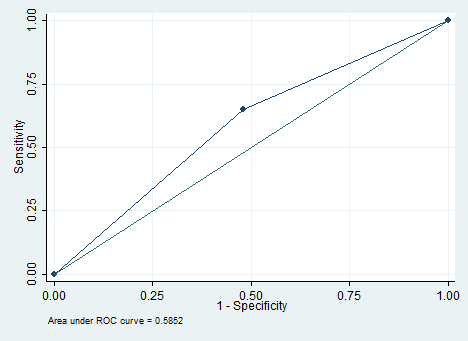


**Usual walking speed (UWS)**

**60-69 years old**


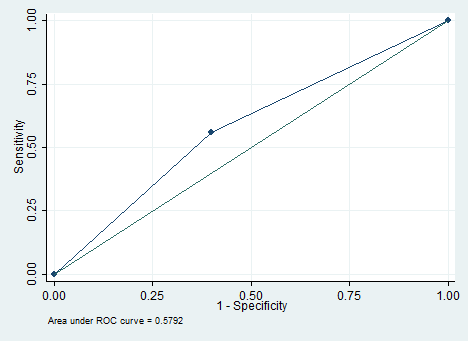

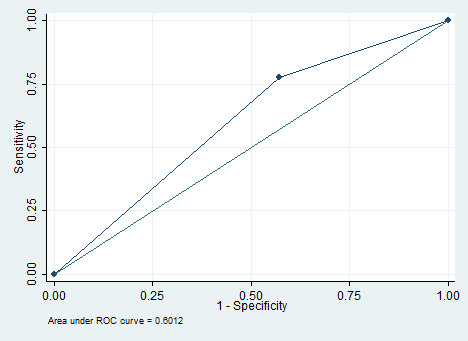


**70-79 years old**


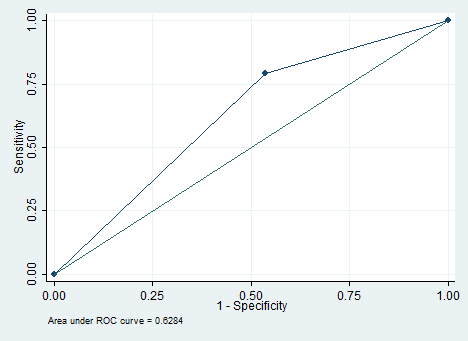

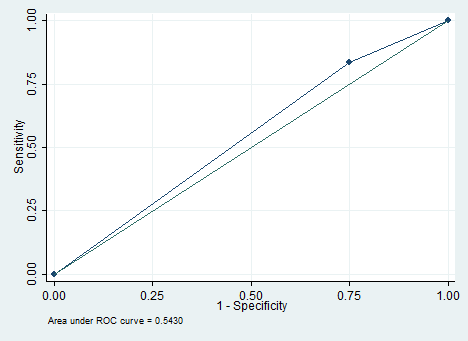


**Overall functions**

**60-69 years old**


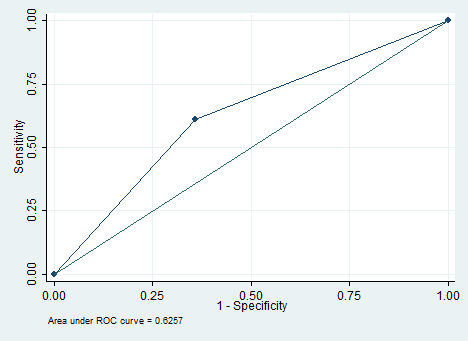

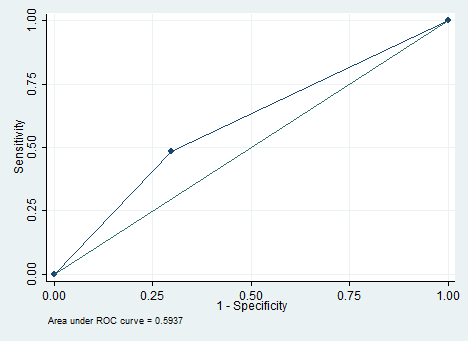


**70-79 years old**

**
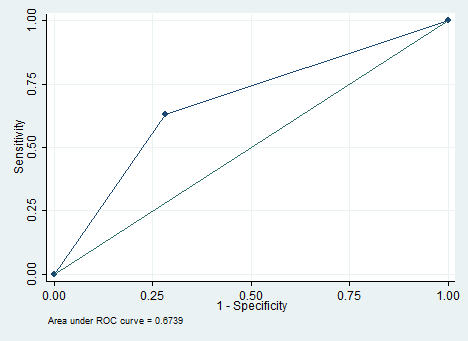

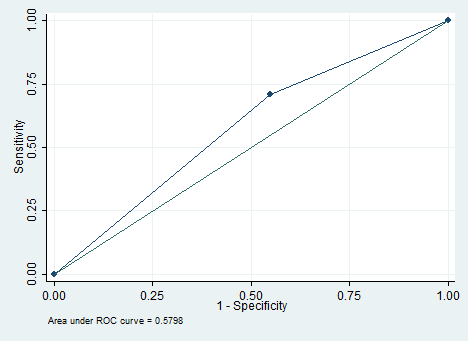
**
